# Are food taxes for healthy eating acceptable? A survey of public attitudes in the UK

**DOI:** 10.1136/bmjph-2024-001731

**Published:** 2025-04-17

**Authors:** Mario Martínez-Jiménez, Hannah Brinsden, Franco Sassi

**Affiliations:** 1 Centre for Health Economics & Policy Innovation, Department of Economics & Public Policy, Imperial College Business School, Imperial College London, London, UK; 2 The Food Foundation, London, UK

**Keywords:** Public Health, Cross-Sectional Studies, Nutrition Surveys, Sociodemographic Factors, legislation and jurisprudence

## Abstract

**Introduction:**

Appropriately designed food taxes can improve diet quality and health. Fiscal levers are used in several countries to combat the rise in obesity and diet-related diseases. This study aims to investigate public attitudes, knowledge and policy preferences regarding food taxes for promoting healthy eating in the UK.

**Methods:**

A survey was administered through YouGov Plc to a nationally representative sample of 2125 adults, gathering information on: acceptability and support for different types of food taxes, awareness and knowledge of existing taxes and preferences for the characteristics of possible new taxes.

**Results:**

Overall, 48% of respondents support higher taxes on unhealthy foods, rising to 72% if taxes made healthy foods more affordable. Respondents with high socioeconomic status and those living in London showed the highest support. Respondents had limited awareness of existing food and beverage taxes and prioritised discretionary items such as cakes and crisps for possible increased taxation.

**Conclusions:**

The survey shows a high level of support for taxing unhealthy foods, as well as concern for the affordability of healthy foods. A carefully designed holistic approach to food taxation can be part of a wider public health strategy and can be favourably met by the general population in the UK.

**WHAT IS ALREADY KNOWN ON THIS TOPIC**

- Fiscal policies, including taxes on unhealthy foods and sugar-sweetened drinks, can shape dietary habits and lower the intake of unhealthy products, leading to better public health outcomes. The success and longevity of these policies heavily rely on public support and trust in government. Research indicates that associating taxes with health benefits or designating revenues for health programmes enhances public approval.

**WHAT THIS STUDY ADDS**

- This research presents current, nationally representative insights into public attitudes, knowledge and preferences concerning food taxes in the UK. It reveals strong support for food taxes, especially when accompanied by initiatives to make healthy food more affordable. The research also points out sociodemographic variations in support and emphasises particular food categories, like cakes and crisps, for possible taxation, providing actionable recommendations for policymakers.

**HOW THIS STUDY MIGHT AFFECT RESEARCH, PRACTICE OR POLICY**

- This study highlights the need to design food taxes in alignment with public preferences and affordability issues to improve acceptability. The results can guide policymakers in customising fiscal measures to boost public support and effectiveness. This may shape the creation and execution of wide-ranging strategies aimed at reducing diet-related diseases and encouraging healthier eating in the UK. It also lays the groundwork for additional research on the socioeconomic effects and long-term efficacy of these policies.

## Introduction

 The use of fiscal incentives to promote healthy eating has increased in the past decade, amid a continuing growth in obesity and diet-related diseases. While many countries currently apply taxes on sugar-sweetened beverages,^1^ a smaller number of countries have used taxes to promote healthy eating, and most of the latter are limited in scope.^2^ This study aims to provide insights on public attitudes for policymakers seeking to use fiscal levers to address the challenge of unhealthy dietary choices.

Food taxation has the potential to improve health by reducing the consumption of unhealthy foods and improving diet quality.^3^ The ultimate goal of health taxes applied on food is to curb diet-related conditions such as obesity, diabetes and tooth decay.^4^,^6^

Public support for taxes is an important factor for successfully getting a tax implemented,^7^ even more so in the context of the rising cost of living where many people are concerned about food prices.^8^ Understanding public attitudes towards food taxes is essential for the successful implementation and sustainability of such health policies. This study aims to provide valuable information for policymakers, enabling them to design and advocate for food taxation measures that are not only effective in promoting healthier eating habits but also supported and acknowledged by the public. This, in turn, can lead to improved public health outcomes and a reduction in diet-related diseases in the UK.

Our study explores public attitudes towards the implementation of food taxes aimed at promoting healthy eating by conducting a nationally representative survey of 2125 adults in the UK between 12 and 14 April 2024. Our survey covers three main areas: (i) support, in principle, for a new tax on unhealthy food; (ii) awareness of the current taxes on foods and non-alcoholic beverages in the UK, the value-added tax (VAT) and the soft drink industry levy (SDIL); and (iii) public preferences regarding specific characteristics of a possible new tax on unhealthy foods. Our headline results show that there is support for food taxes on unhealthy food, particularly when coupled with measures to make healthier food more affordable. Findings also show poor awareness of existing taxes, which is poorer for the SDIL than for VAT. Support is higher among females, people of high social class and people living in London. Priority targets for possible taxes on unhealthy foods included food groups such as cakes, potato crisps, hot takeaways and ready meals.

### Literature review

Food taxation has been extensively studied as a potential public health measure aimed at incentivising healthier dietary choices. Research indicates that prices and promotions within the food environment can significantly influence consumer behaviour, often encouraging the selection of foods contributing to less healthy diets. Taxation of unhealthy products, such as tobacco, alcohol or sugar, is not new, but traditionally, these taxes have been used for purely fiscal reasons, that is, to generate tax revenues in order to finance public spending.

Empirical evidence from various countries demonstrates that taxation can be an effective tool for promoting healthier dietary choices.^9 10^ Reviewing the evidence in 2015, Niebylski *et al*^11^ showed that combined subsidies and taxes of 10%–15% effectively influence dietary choices, while Wright *et al*^12^ later found that a 20% tax rate was needed to achieve significant change. Scarborough *et al*^13^ show that in 2016, as a result of the SDIL—a tax on soft drinks that contain more than 5 g of sugar per 100 mL—the percentage of drinks with sugar fell from 49% to 15% between September 2015 and February 2019 in the UK. Taillie *et al*^14^ show a 12% reduction in sales in the first year following the implementation of Mexico’s 8% non-essential energy-dense food tax, driven by lower-income households. Similarly, Berkeley’s sugar-sweetened beverages (SSB) tax resulted in a 21% drop in the consumption of sugary drinks, demonstrating the potential of such taxes to alter consumer behaviour. In October 2011, Denmark implemented a €2.14 per kg tax on saturated fat for products with more than 2.3 g per 100 g of saturated fat. Although this tax was subsequently repealed in January 2013, for the 15-month duration of implementation, saturated fat purchases were reduced by 4%, and deaths attributable to non-communicable diseases were estimated to have been reduced by 0.4%.^15^ Hungary has introduced a tax targeting prepacked foods that are high in salt, sugar or caffeine (at varying tax rates), which has been associated with a 3.4% reduction in the consumption of processed food (and a compensatory 1.1% increase in unprocessed food) as shown by Bíró *et al*.^16^ However, Thow’s *et al*^17^ systematic review found that subsidies had variable effects on calorie intake, with combined subsidies and taxes of 10–20% leading to a 1% reduction in some studies, while others showed increases of 1–17%, influenced largely by industry pricing strategies.

Public attitudes towards taxes, particularly those aimed at improving health, vary widely depending on the context. In the case of health taxes, such as those on tobacco^18 19^ and sugary drinks,^20^,^23^ there tends to be a higher level of support when the public perceives a clear link between the tax and health benefits. In contrast, general taxes are often viewed through a lens of economic impact and fairness.^24 25^ Public trust in government plays a significant role in shaping these attitudes; higher trust correlates with greater acceptance of both specific and general taxes. Wright *et al*^12^ concluded that commitments to ring-fencing revenue for health must be followed by the government to maintain public support and that framing the goal of a health tax was essential to reduce the possibility of ‘hostile lobbying’. Eykelenboom *et al*^26^ conducted a systematic review synthesising the existing literature on the political and public acceptability of an SSB tax covering countries around the world (eg, the USA, Australia, the UK, Mexico, China, France and New Zealand, among others). The authors found that of the public, 42% supported a tax on SSB, 39% supported an SSB tax as a means of tackling obesity and 66% supported such a tax where the revenue was used ‘appropriately’, for example, for health initiatives. Siegerink *et al*^27^ examined Dutch attitudes towards a meat tax, noting a preference for revenue to be allocated for specific uses, particularly if it lowered taxes on fruits and vegetables. In contrast, Hagmann *et al*^28^ find that only 37.2% of Swiss support a national sugar tax, with preferences for interventions such as labelling and notable differences in acceptability by urban and rural areas and by those most at risk, such as being overweight.

Regarding the acceptability of food taxes, Mazzocchi *et al*^29^ found that the main drivers of policy support in five European countries are attitudinal factors, particularly the belief that obesity is largely due to the excessive availability of unhealthy foods. Sociodemographic characteristics and political preferences, however, are not strongly correlated with support for these policies. In Germany, Jurkenbeck *et al*^30^ found that a large majority of citizens accept nutrition policy interventions, mostly among those individuals who generally maintain a healthy diet and those who struggle with dietary habits. In Norway, Grimsrud *et al*^31^ demonstrated that there is significant public acceptance and willingness to pay for cost-effective taxes, particularly on red meat, despite the public’s scepticism towards the introduction or increase of taxes aimed at environmental improvement. This suggests that while environmental taxes may face resistance, targeted nutritional interventions addressing public health concerns can receive substantial support across diverse demographic groups. Claudy *et al*^32^ point out that taxes are not a standalone measure but work best as part of a package, including front-of-pack labelling, advertising restrictions and educational campaigns.

Our study builds on previous research by providing updated, nationally representative insights into public attitudes, knowledge and preferences regarding food taxes in the UK. While earlier studies have explored the effectiveness of food taxes in improving diet quality, our research uniquely contributes to a deeper understanding of how fiscal policies can be tailored to maximise public acceptance.

## Materials and methods

### Survey data collection and sample

We collected our survey data between 2 and 14 April 2024 through YouGov Plc (https://yougov.co.uk/). The survey was conducted through an online questionnaire sent to members of the YouGov panel, which consists of 185 000+ individuals who have agreed to participate in the Company’s surveys. To ensure the sample is representative of the UK population or specific subgroups, stratified sampling techniques are used to select respondents based on key demographics such as age, gender, region and socioeconomic status. Surveys are distributed to the selected sample via email invitations or through the YouGov platform, allowing respondents to respond at their convenience. YouGov typically achieves a response rate of between 35% and 50% for surveys, although this can vary depending on the subject matter, complexity and length of the questionnaire. Further details regarding the survey methodology and questionnaire are available in online supplemental Appendix A.1–A.2.

The responding cross-sectional sample is weighted to match the profile of the original target sample. During the survey period, response rates and data quality are monitored, and if certain demographics are underrepresented, additional invitations may be sent to those groups. The survey was completed by 2125 adults between 12 and 14 April 2024, using an online questionnaire. Table 1 shows that the resulting (weighted) sample is representative of the UK population. One dimension in which our sample may differ is regarding political preferences (captured by the 2019 General elections and EU referendum in 2016), in which missing observations account for around 26 and 23 per cent of our sample (see Table A2 in online supplemental Appendix A.3). For that reason, political party preference is not used in our main analysis. All analyses reported in this paper are based on the weighted sample.

**Table 1:** Sample representativeness and summary statistics of survey participants

	Weighted sample		Unweighted sample		Population%
	N	%	N	%	
Gender					
Male	1031	48.5	966	45.5	49.45
Female	1094	51.5	1159	54.5	50.55
Age					
18–24	223	10.5	192	9	8.3
25–49	878	41.3	894	42.1	32.9
50–64	527	24.8	534	25.1	19.5
65+	497	23.4	505	23.8	18.4
Social grade					
ABC1	1211	57	1258	59.2	57
C2DE	914	43	867	40.8	43
Country					
England	1785	84	1790	84.2	84
Wales	104	4.9	113	5.3	4.7
Scotland	181	8.5	172	8.1	8.2
Northern Ireland	55	2.6	50	2.4	2.9
Region in England					
North	499	27.9	507	28.3	27
Midlands	342	19.2	354	19.8	20
London	251	14.1	217	12.1	16
South	693	38.9	712	39.8	32
Total	2125	100	2125	100	100

Total sample size is 2125 adults. Fieldwork was undertaken between 12 and 14 April 2024. The survey was carried out online. The figures have been weighted and are representative of all UK adults (aged 18+). *Source:* YouGov and Office National Statistics (ONS).

### The questionnaire and demographic characteristics

The questionnaire is structured in three parts, described as (*i*) acceptability/support, (*ii*) awareness/knowledge and (*iii*) preferences on characteristics of a possible food tax. Our questionnaire includes eight closed-ended questions. Three questions were asked about awareness/knowledge of the UK tax system for food and drinks (VAT and SDIL), and two questions addressed the acceptability of taxes on unhealthy food. Finally, a set of questions captures individuals’ preference for a possible new tax on unhealthy foods. This refers to one question focused on which food groups should or should not have a higher tax, while two more questions asked about specific goals, such as discouraging people from purchasing certain products and encouraging them to buy others instead.

Based on previous public opinion research on perception and attitudes towards taxes and policies, we asked respondents about their basic socioeconomic and demographic characteristics, including their age (<30; 30–44; 45–64; >64) and social class (‘grade A, professionals; very senior managers business; top-level civil servants’, ‘grade B, middle-management executives/principal officers/top management or owners of small business‘ or ‘grade C1, junior management/varied responsibilities and educational requirement’ and zero if ‘grade C2: skilled manual workers/manual workers with responsibility for other people’, ‘grade D, semiskilled and unskilled manual workers, apprentices and trainees of skilled workers’ or ‘grade E, long-term recipients of state benefits/unemployed/off sick/casual workers’). We also controlled for region and country (London, Scotland, Wales, Northern Ireland and England). More information on the variables is provided in online supplemental Appendix A.2.

Our study measures respondents’ support for food taxes that promote healthy eating through two main questions: ‘would you support or oppose a higher tax on unhealthy foods?’ and ‘would you support a higher tax on unhealthy foods if money makes healthy food cheaper?’.

### Statistical approach

To investigate the relationship between respondent characteristics and support for food taxes towards healthy eating habits, we estimate a logistic model using the statistical software Stata 18.5 (see more information on the empirical methodology in online supplemental Appendix B.1). A set of outcome variables captures to what extent a tax on unhealthy foods is supported by respondents. These variables are binary regarding the extent of support for taxes on unhealthy food, taking values one if the respondent ‘strongly/somewhat supports’ and zero if the respondent ‘strongly/somewhat opposes’. It is important to note that ‘don't know’ observations are not accounted for in the main statistical analysis (final N is 1971 for tax on unhealthy food, and 1995 for tax on unhealthy food to make healthier food cheaper). This means that the statistical analysis focuses only on the responses where a clear opinion was provided, providing a clearer picture of the attitudes and preferences of the respondents who expressed a definite opinion.

Sociodemographic characteristics included in the analysis are age groups, a binary variable capturing manual/non-manual social class (which takes one if the respondent’s social class grade is A/B/C1 and zero if C2/D/E). We also controlled by regions/countries. We provide estimates with robust standard errors that account for potential violations of model assumptions, particularly heteroskedasticity and certain forms of model misspecification (eg, the functional form of the covariates or omitted variables). Survey weights are used in all the regressions. Sensitivity analyses are performed, including using alternative statistical models (including a linear probability model and ordered logit model), controlling for political preferences and levels of self-reported knowledge of food taxes and testing for multicollinearity bias when selecting our control variables. Results for all the sensitivity analyses are available in Tables A2 and A5 in online supplemental Appendix A and Figures B1–B3 in online supplemental Appendix B.2.

## Results

### Knowledge of existing taxes applied to food and non-alcoholic drink products

We first analyse the level of awareness of existing taxes applied to foods and non-alcoholic beverages, VAT and SDIL. We found greater awareness of VAT compared with SDIL, with just 4% considered to have ‘little knowledge’ of VAT, compared with 23.2% for SDIL. There was, however, a similar number of people who we classify as ‘very knowledgeable’ across both of the taxes (27.4% for VAT and 25.6% for SDIL)—see Figure C1 in online supplemental Appendix C.

We then asked whether a selection of foods has or does not have SDIL and VAT applied to them. While 68% correctly identified that soft drinks have VAT, only 28.9% identified that bottled water had VAT applied. There is also high awareness among respondents that foods from both takeaways (69%) and restaurants (78.8%) have VAT applied. More than half of respondents (57.5%) incorrectly thought that cakes had VAT applied, with only 18.9% correctly identifying that they did not. The responses on the two meat options (fresh and processed) were mixed, with more people thinking VAT is applied to processed meat compared with fresh meat, 40% correctly identifying that fresh meat does not have VAT, and only 24.4% correctly identifying that processed meat does not. Between 14% and 30% of respondents reported they do not know whether taxes apply for each of the mentioned food groups, with the highest rate for bottled water and the lowest rate for restaurant meals—see Figure C2 in online supplemental Appendix C.

### Support and opposition for higher food taxes for healthier eating habits

Overall, 48% of respondents supported higher food taxes (compared with 44% who opposed them), and this number would increase to 72% if the money raised was used to make healthier food more affordable. Only 23% opposed higher taxes on unhealthy foods if the revenue generated was used to make healthier food cheaper.

**Figure 1:**
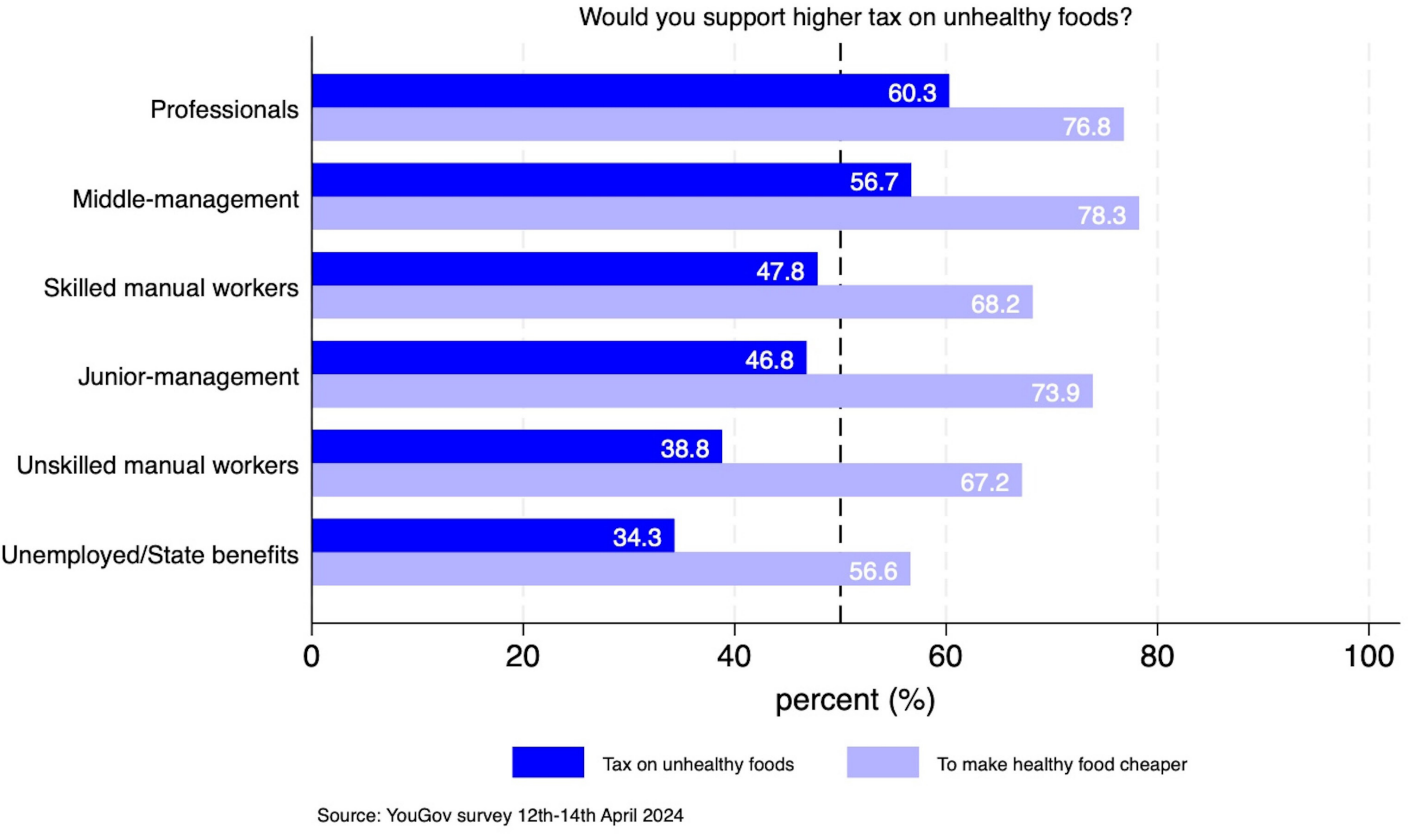
Share of respondents who agree (somewhat too strongly) that ‘would you support a higher tax on unhealthy foods?’ or ‘would you support a higher tax on unhealthy foods to make healthy food cheaper’ by socioeconomic status. The number of observations is 2,125; the base category includes the following responses: strongly/somewhat opposed/don’t know. The support category includes responses that strongly/somewhat support. Socioeconomic status groups include the following professions: professionals (grade A: professionals; very senior managers in business; top-level civil servants); middle-management (grade B: middle-management executives/principal officers/top management or owners of small businesses); junior-management (grade C1: junior management/varied responsibilities and educational requirements); skilled manual workers (grade C2: skilled manual workers/manual workers with responsibility for other people); unskilled manual workers (grade D: semi-skilled and unskilled manual workers, apprentices and trainees of skilled workers); and unemployed/state benefits (grade E: long-term recipients of state benefits/unemployed/off sick/casual workers). Sample weights are used.

Regarding respondents’ level of support for food taxes on unhealthy food by sociodemographic characteristics, we find no substantial variation in support by age group: 18–24 (49 vs 42%), 25–49 (46 vs 45), 50–64 (50 vs 45) and 65+ (50 vs 44), as we can see in Figure C3 in online supplemental Appendix C. However, some differences are found by socioeconomic status. In figure 1, we can see that people in non-manual professions are more likely to support higher taxes on unhealthy food, with percentage ranges of support between 60% and 48%. In contrast, those individuals who are long-term recipients of state benefits, unemployed or casual workers show the lowest support for these taxes with 34% of respondents. We conducted a Bonferroni post hoc analysis of variance (ANOVA) to assess differences in support for taxing unhealthy food across professional categories. Significant differences were found between high-skilled professions compared with those in lower occupational categories (see these results in Table C1 and C2 in online supplemental Appendix C).

By political party preferences in the 2019 General Elections, we can see that those who voted for left-oriented parties have higher support for a tax on unhealthy foods (eg, support for a tax on unhealthy foods was 58.9% among former labour voters vs 49.2% among former conservative voters; see Figure C4 in online supplemental Appendix C for complete results). By region and country, we find the highest support in London, with 54% of respondents supporting a tax on unhealthy food, increasing to 76.7% if the tax was used to make healthier food more affordable. Respondents living in Wales had the lowest support for the tax at 37.8%. Northern Ireland had the biggest increase in support when asked about making healthier food more affordable, with an increase from around 44% to 74%. The lowest support in England was among those living in the North East and West Midlands, with 44.6% and 44.5% reporting to support a tax on unhealthy food, respectively (see Figure C5 in online supplemental Appendix C).

**Figure 2:**
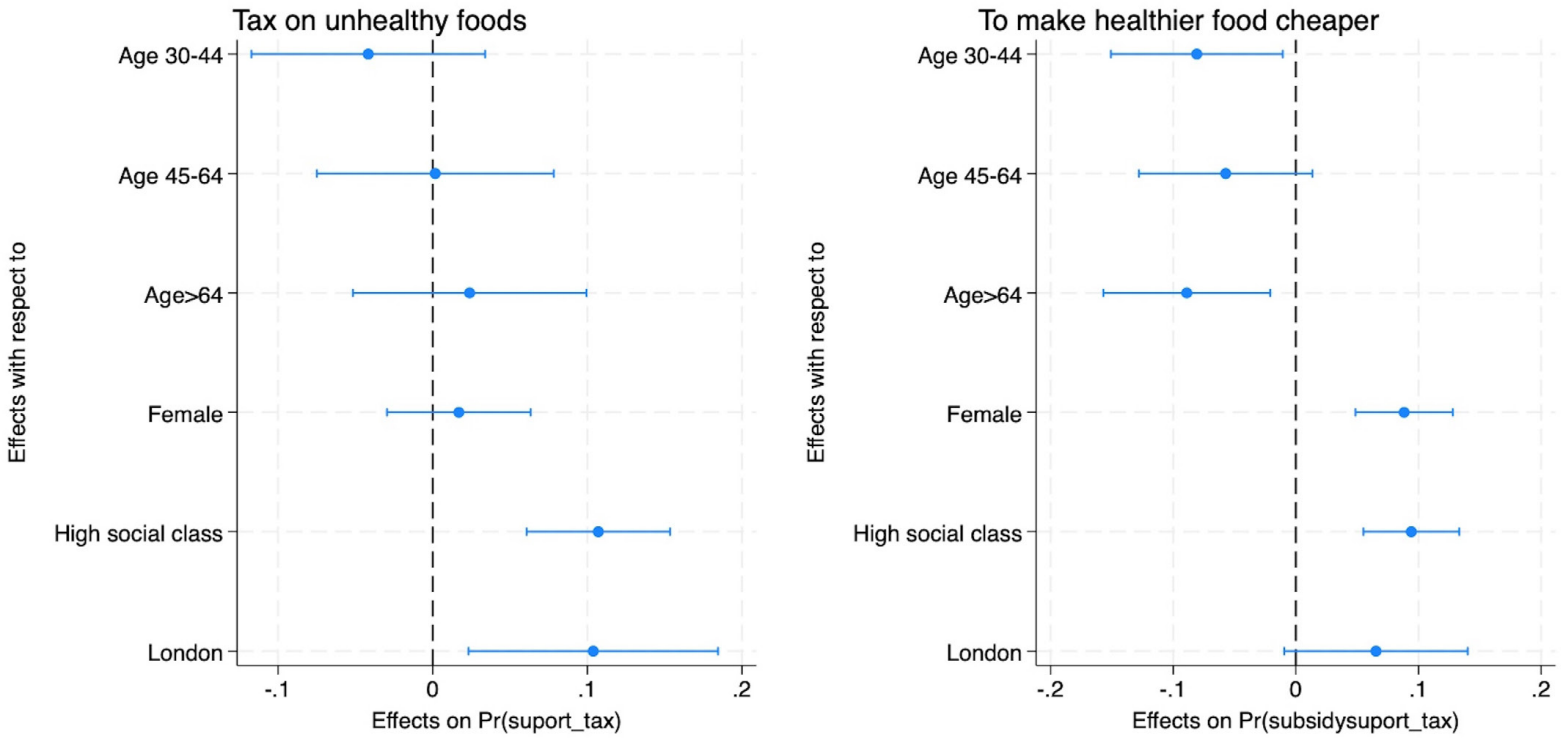
Logistic regression estimates showing the correlation between support for a tax on unhealthy foods and socioeconomic characteristics. Outcome 1 (first column): tax on unhealthy. Outcome variable 2 (second column): tax on unhealthy food to make healthier food cheaper. Binary outcome variables take one if they somewhat support or strongly support and zero if they somewhat or strongly oppose. The number of observations is 1971 (outcome 1) and 1995 (outcome 2), as those answering ‘do not know’ are not included in both outcome variables. Average marginal effects estimates (green dots) are shown. Sample weights are used, and confidence intervals (green lines) are plotted. Base categories: age between 18 and 30; male; manual professions (social class grade: C2/D/E); other countries and English regions of the UK.

Figure 2 shows the results of the logistic regression analysis of supporting taxes on unhealthy foods conditional to certain socioeconomic characteristics (see more statistical information in Table C3, online supplemental Appendix C). In particular, we control the support on food taxes on age groups (reference group is those between 18 and 30 years old), gender, non-manual social class and living in London. Here, we can see that those in non-manual professions and living in London are statistically significantly more likely to support taxes on unhealthy foods compared with their counterparts. Similarly, women and those in non-manual professions are more likely to support a tax on unhealthy food that makes healthy food cheaper.

### Insights on preferences for policy design and purpose

Table 2 shows levels of support for a possible tax on unhealthy foods for the full sample and specifically for those who had reported supporting or opposing taxes on unhealthy foods. Overall, we can see a similar pattern of support when comparing the full sample to just those who supported a tax, with higher support and lower opposition seen for specific categories among those who supported a tax. A much bigger difference between support and opposition was also seen within the smaller sample of supporters.

**Table 2:** Support for food taxes by food categories and level of support for a tax on unhealthy foods

Degree of support for a tax on unhealthy foods	Food category							
	Milkshakes	Fruit juice	Fresh fruit and vegetables	Potato crisps	Read and processed meat	Cakes	Ready meals	Hot takeaways and deliveries
Full sample (n=2125**)**								
Support	32.66	11.56	1.37	40.87	22.92	44.68	36.20	39.51
Oppose	52.01	75.60	91.41	45.99	64.62	41.96	49.96	47.09
Do not know	15.32	12.84	7.22	13.14	12.46	13.36	13.84	13.40
Support a tax (n=1047**)**								
Support	51.73	18.95	1.72	65.13	37.78	68.73	56.55	60.22
Oppose	35.78	69.67	93.98	24.05	51.42	21.14	32.31	29.20
Do not know	12.50	11.39	4.51	9.82	10.79	10.13	11.14	10.58
Oppose a tax (n=924**)**								
Support	15.04	4.64	0.86	18.50	8.15	22.22	17.38	20.18
Oppose	73.32	86.90	94.37	71.67	83.95	67.29	72.64	69.67
Do not know	11.63	8.46	4.77	9.83	7.90	10.49	9.98	10.15
Don’t know* (n=154**)**								
Support	15.01	5.23	1.06	18.07	14.95	23.74	17.39	21.39
Oppose	32.25	49.03	61.84	30.47	37.41	27.60	31.18	30.27
Do not know	52.72	45.74	37.10	51.46	47.62	48.66	51.43	48.33

Total sample size is 2125 adults. Fieldwork was undertaken between 12 and 14 April 2024. These results come from the following question: ‘do you think the following foods should or should not have a higher tax applied to them?’ and include average sample weights

* ‘Don’t know’ could mean the person does not know/have a view, does not understand the question or does not want to answer the question.

n, number of observations.

The only category which had more support than opposition among the full sample was cake, with 44.68% reporting to support a potential tax on this category (vs 42.12% who opposed it). This figure increased to 68.73% support (and 21.14% opposed) when only looking at those who were generally supportive of a tax. The second most supported category was potato crisps (40.87% support vs 45.99% opposed); however, again, support increased to 65.13% (vs 24.04%) when looking only at those who supported a tax. Other categories with support include hot takeaways (39.51%) and ready meals (36.20%). Support was low for a tax on red and processed meat (23.29%) and, unsurprisingly, fruit and vegetables (1.37%). Overall, we can see more support for a tax on discretionary products which fall outside of the Government recommended Eatwell Plate (eg, cakes and biscuits) rather than meals (ready meals and takeaways) and less processed foods (eg, fruits, vegetables and meat).

**Figure 3:**
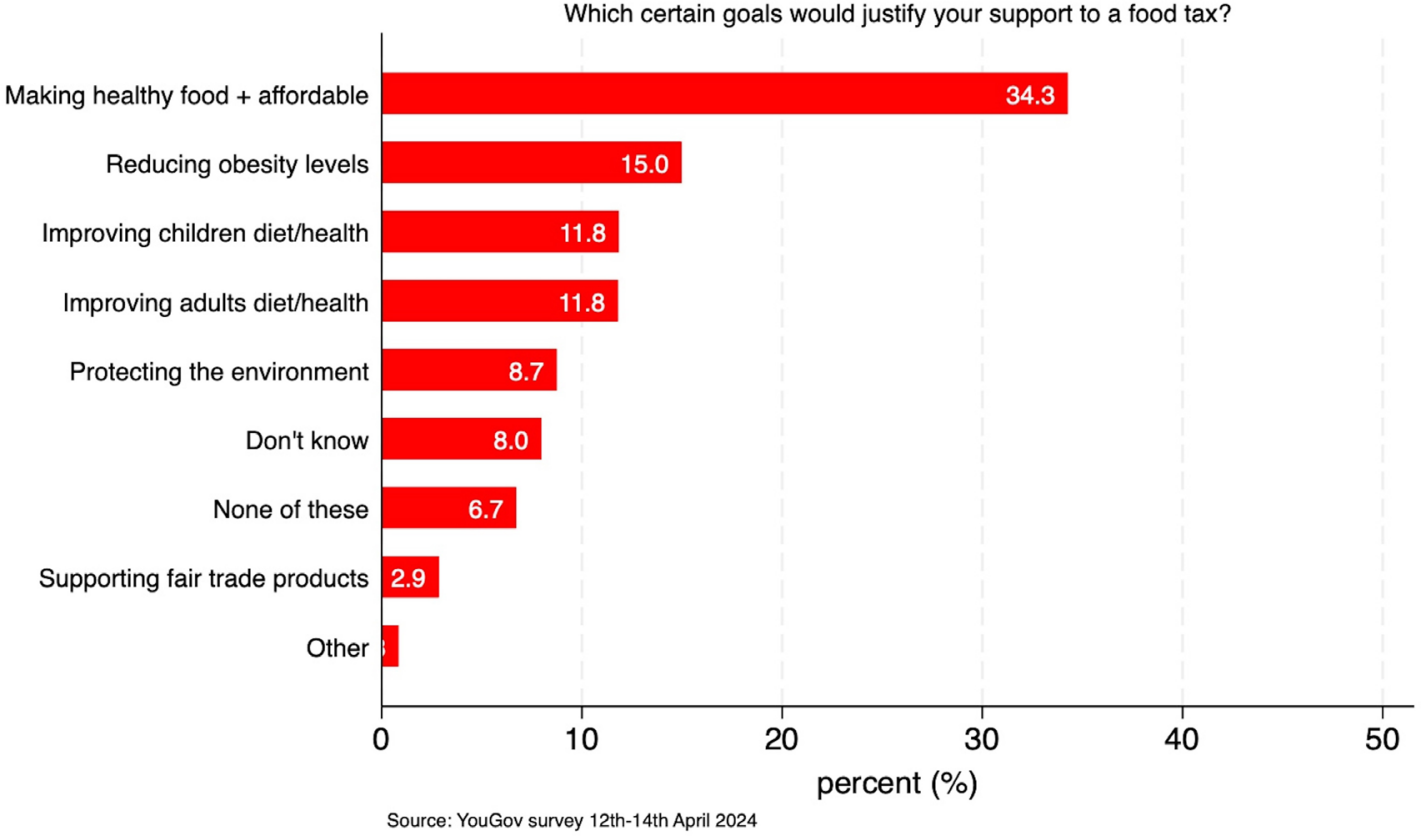
Share of respondents on each of the goals that could be tackled by taxing food and drink products. The total sample size is 2125 adults. Question 8: ‘some taxes are applied to products to specifically achieve certain goals, either through the revenue raised or by discouraging people from purchasing those products and encouraging them to purchase others instead. Thinking about different goals that could be tackled by taxing food and drink products, which of the following do you think is most important?’

To better understand what criteria/outcomes respondents most value to justify a tax on foods, respondents were asked about their priority goal that a tax should address. As we can see in figure 3, the highest support of the options provided was for making healthier food more affordable (34.3%), followed by obesity (15%) and improving children’s and adults’ diet and health (both at 11.8%). Fewer people put fair trade and environmental outcomes as their priority. The high support for a tax which can make healthier food more affordable is consistent with the findings that there is greater support for a tax if it is also used to make healthier food more affordable. It is also reflective of the current cost-of-living crisis, which has led to one in five households reporting to be affected by food insecurity in January 2024^33^ and food prices increasing by roughly 25%.^34^ These results should be interpreted with caution due to a potential priming effect, derived in particular from the framing of the initial questions on the desirability of taxes pursuing specific policy goals (eg, making healthy foods less expensive) which may have inflated support for certain options. Figures C6 and C7 in online supplemental Appendix C show less opposition for a tax that would make healthy food groups (eg, fruits) cheaper and unhealthy food groups (eg, cakes) more expensive compared with a tax making healthy option within food group cheaper and unhealthy more expensive (eg, making cheaper *healthy* ready meal options and more expensive *unhealthy* ready meal options).

## Discussion and policy implications

Our representative national survey of 2125 people in the UK provides valuable insights into public attitudes towards food taxes that promote healthier eating. The findings highlight several key aspects that policymakers need to consider when designing and implementing such taxes.

While the survey indicates considerable support for taxing unhealthy foods, the level of this support varies among different groups of respondents. Support for the tax idea is significantly higher when paired with efforts to make healthier food more affordable. This finding is consistent with those reported in other studies of the acceptability of sugar-sweetened beverages (SSBs)^26^ and food^27^ taxes and has far-reaching policy implications. The public is prepared to accept health taxes when the health rationale is clear, but also when taxes are not merely designed to make unhealthy consumption more expensive. Support tends to be conditional on the use that a government intends to make of the revenues collected through the tax or tax hike. In the case of food, affordability is a primary concern for many consumers, especially at a time of high food inflation such as that experienced by respondents to our survey. To ensure that the affordability of an adequate food basket is not compromised by a tax on less healthy foods, the public rightly expects that healthier foods are made more affordable, as part of a package of fiscal measures designed to improve diet quality. The fact that support by respondents of lower socioeconomic status tends to be more cautious likely underscores affordability concerns.

The survey also reveals that a substantial number of respondents lack adequate knowledge about the taxes currently imposed on food and non-alcoholic beverages in the UK. This lack of awareness regarding the health impacts of unhealthy foods and the potential benefits of food taxes may significantly influence public attitudes towards these taxes. Respondents demonstrate differing preferences concerning which specific foods should be taxed, the goals of the tax and the distribution of tax revenues. Notably, there is a preference for taxing discretionary items like cakes and crisps rather than meals, with revenues aimed at making healthier food more affordable. Consistent with preferences described above, the objective for a food tax that was supported by the largest number of respondents to our survey is to enhance the affordability of healthier foods. Recognising these preferences can assist policymakers in designing food taxes that are more likely to receive public backing and achieve the intended health impacts.

Our study has a few limitations. First, while YouGov aims to ensure representative sampling, online panels may exclude individuals without internet access, and the reliability of weighting and statistical adjustments relies on the accuracy of external benchmarks (such as census data). Second, respondents were not given detailed information regarding the potential scope or extent of the tax; therefore, support levels might vary in response to a comprehensive proposal from policymakers.

Overall, our study provides evidence that carefully designed food taxes aimed at incentivising healthy eating and curbing diet-related diseases can be supported by a sizeable majority of the UK population, provided that attention is paid to the affordability of healthy foods when designing the tax incentives.

## Supplementary material

10.1136/bmjph-2024-001731 online supplemental file 1

## Data Availability

Data may be obtained from a third party and are not publicly available.
