# Supplementary material for: Are food taxes for healthy eating acceptable? A survey of public attitudes in the UK
Source: BMJ Public Health. 2025 Apr 17;3(1):e001731. doi: 10.1136/bmjph-2024-001731 (PMC12010336; doi:10.1136/bmjph-2024-001731)
Supplement: online supplemental file 1 [file bmjph-3-1-s001.docx]

**SUPPLEMENTARY APPENDIX**

**Appendix A. YouGov dataset, questionnaire and variables definitions**

**A.1. YouGov survey methodology**

The survey was conducted online via YouGov's proprietary panel. Panel members are invited to take part in a survey via email or the YouGov App.^[[1]](#footnote-2)^All the details about our methodology are on the website (<https://yougov.co.uk/about/panel-methodology/>). Consent to participate in surveys and for YouGov to collect and hold their data is obtained when respondents first join the YouGov panel. There was no additional, specific consent section for this study. The total sample size was 2,125 adults. Fieldwork was undertaken between 12th - 14th April 2024. The survey was carried out online. Our analyses have been weighted and are representative of all UK adults (aged 18+).

YouGov cannot determine the average completion time for our survey, as it was fielded on the Political Omnibus service. This is because when YouGov process the data, they are only giving completion times for the entire Omnibus, not individual client surveys. Our section of the survey was a total of 11 units, shown across 8 pages. However, it was part of an omnibus service, where the total number of pages was c.50. The maximum number of minutes the median survey completion time would be is 16.

This survey has been conducted using an online questionnaire administered to members of the YouGov Plc GB panel of 185,000+ individuals who have agreed to take part in surveys. An email was sent to panellists selected at random from the base sample according to the sample definition, inviting them to take part in the survey and providing a link to the survey. (The sample definition could be "GB adult population" or a subset such as "GB adult females"). YouGov does not provide an exact response rate for individual surveys due to the type of sampling employed. YouGov uses active quota sampling, so YouGov does not invite a specific 2000 people to take the survey - the email panellists receive just brings them into their system, not into that exact survey. From there, YouGov has an algorithm system which takes them to the survey that needs a person with those characteristics most, based on the quota frame associated with the surveys in the field at any one time. There will be numerous surveys in the field at any moment. However, YouGov typically achieves a response rate of between 35% and 50% to surveys, though this does vary depending on the subject matter, complexity and length of the questionnaire. The responding sample is weighted to the profile of the sample definition to provide a representative reporting sample. The profile is normally derived from census data or, if not available from the census, from industry accepted data. YouGov makes every effort to provide representative information. All results are based on a sample and are, therefore subject to statistical errors normally associated with sample-based information.

YouGov conducts identity and fraud controls at recruitment and survey entry. They also conduct post-hoc cleaning of the data. Measures taken include: System Level (Geolocation/IP, Red flags on suspect behaviours), Welcome Survey Level (Double opt-in, Email Domain Check, Country of Residence vs IP (COR-IP), Data point consistency), and In-Survey Level (Soft launch checks, Quality trap questions, Identifier QC, Attention QC, Inattentiveness scanning, Hyperactiveness scanning, Speed monitoring).

**Selection bias and sampling error in YouGov surveys**

YouGov employs several strategies to address selection bias and sampling error in its survey data to ensure representative and reliable results. Selection bias arises when survey respondents do not represent the population of interest. YouGov mitigates this through the following:

1. Large and Diverse Online Panel: YouGov maintains a large, diverse panel of respondents recruited from various sources. This panel reflects the demographic and attitudinal diversity of the population.
2. Quota Sampling: Respondents are selected using quotas based on key demographic variables such as age, gender, region, and education level. Quotas ensure the sample includes proportional representation of different subgroups.
3. Weighting: YouGov applies post-stratification weights to adjust for any imbalances in the sample.
4. Weights are based on known population characteristics (e.g., census data) to align the sample with the target population.
5. Frequent Validation: Panel data are continuously validated against external benchmarks, such as election results or demographic statistics, to monitor and correct biases.

Sampling error occurs when the selected sample does not perfectly represent the population. YouGov addresses this by:

1. Random Sampling within Panels: Respondents for each survey are randomly selected from the panel within demographic quotas, reducing systematic errors.
2. Representative Sampling via Statistical Matching: YouGov uses multilevel regression and post-stratification (MRP) for certain surveys, a technique that combines survey data with external datasets to estimate attitudes across different population subgroups.
3. Sample Size: They often use larger sample sizes than traditional polling to minimize sampling error, especially for subgroup analysis.

Our analyses use weighting adjustments also help to account for underrepresented groups due to differential nonresponse.

**A.2. Questionnaire and variables definitions**

**Q1) Generally speaking, would you support or oppose a higher tax on unhealthy foods?**

<1> Strongly support

<2> Somewhat support

<3> Somewhat oppose

<4> Strongly oppose

<5>Don’t know

{single}

**Q2) And would you support or oppose a higher tax on unhealthy foods if the money raised was used directly to help make healthier food cheaper?**

<1> Strongly support

<2> Somewhat support

<3> Somewhat oppose

<4> Strongly oppose

<5>Don’t know

[grid]

**Q3) How much, if at all, do you know about the following taxes and which foods they are added to?**

- **Value Added Tax (VAT)**
- **Soft Drink Industry Levy (SDIL)**

<1> I know what it is, and the foods it is added to

<2> I know what it is, but know little about what foods it is added to

<3> I have heard of it, but don't know anything about what foods it is added to

<4> I have never heard of it and don’t know anything about what foods it is added to

[grid]

**Q4) Which of the following foods do you think have and don’t have VAT added to them?**

1. Soft drinks
2. Bottled water
3. Fresh meat
4. Processed meat (e.g. ham)
5. Cakes
6. Chocolate and confectionery
7. Food eaten in restaurants and cafes
8. Hot takeaways and deliveries

<1> Does have VAT

<2> Does not have VAT

<3> Don’t know

**Q4) Which of the following drinks do you think have and don’t have Soft Drinks Industry Levy (SDIL) added to them?**

1. Soft drinks
2. Bottled water
3. Fruit juice
4. Milkshakes

<1> Does have SDIL

<2> Does not have SDIL

<3> Don’t know

[grid]

**5) Do you think the following foods should or should not have a higher tax applied to them?**

1. Milkshakes
2. Fruit juice
3. Fresh fruit and vegetables
4. Potato crisps
5. Red and processed meat
6. Cakes
7. Ready meals
8. Hot takeaways and deliveries

<1> Should have a higher tax

<2> Should not have a higher tax

<3> Don’t know

{Single}

**Q6) Some taxes are applied to products to specifically achieve certain goals either through the revenue raised or by discouraging people to purchase those products and encouraging them to purchase others instead.**

**Thinking about different goals that could be tackled by taxing food and drink products, which of the following do you think are most important?**

<1> Protecting the environment

<2> Improving adults’ diet and health

<3> Supporting fair trade products

<4>Making healthy food more affordable

<6> Improving children’s diet and health

<7> Reducing obesity levels

<8> Other

<9> None of these

<10> Don’t know

{grid}

**Q7) Would you support or oppose the following…**

- **Making it less expensive for people to choose healthy foods (e.g. fruit and vegetables), and more expensive to choose less healthy foods (e.g. deserts, prepared meals)**
- **Making it less expensive for people to choose healthier options within food groups (e.g. ready meals containing less fat, salt and sugar) and more expensive to choose less healthy options within the same food group**

<1> Strongly support

<2> Somewhat support

<3> Strongly oppose

<4> Somewhat oppose

<5> Don’t know

**Table A1.** YouGov variables description

| **Variables** | **Description** |
| --- | --- |
| age | Age |
| profile_gender | Gender |
| profile_socialgrade_cie | Social Grade (Chief Income Earner) A / B / C1 / C2 / D / E |
| profile_GOR | Region lived |
| voted_ge_2019 | Talking to people about the General Election in December 2019, we have found that a lot of people didn’t manage to vote. How about you – did you manage to vote in the General Election? |
| pastvote_ge_2019 | Which party did you vote for at the General Election in December 2019? |
| pastvote_EURef | Past vote 2016 EU referendum |
| Weight | Weight |
| FF1 | Generally speaking, would you support or oppose a higher tax on unhealthy foods? |
| FF2 | And would you support or oppose a higher tax on unhealthy foods if the money raised was used directly to help make healthier food cheaper? |
| FF3 | How much, if at all, do you know about the following taxes and which food and drinks they are added to? |
| FF3_1 | Value Added Tax (VAT) |
| FF3_2 | Soft Drink Industry Levy (SDIL) |
| FF4 | Which of the following foods do you think have and don’t have VAT added to them? |
| FF4_1 | Soft drinks |
| FF4_2 | Bottled water |
| FF4_3 | Fresh meat |
| FF4_4 | Processed meat (e.g. ham) |
| FF4_5 | Cakes |
| FF4_6 | Chocolate and confectionery |
| FF4_7 | Food eaten in restaurants and cafes |
| FF4_8 | Hot takeaways and deliveries |
| FF5 | Which of the following drinks do you think have and don’t have Soft Drinks Industry Levy (SDIL) added to them? |
| FF5_1 | Soft drinks |
| FF5_2 | Bottled water |
| FF5_3 | Fruit juice |
| FF5_4 | Milkshakes |
| FF6 | Do you think the following foods should or should not have a higher tax applied to them? |
| FF6_1 | Milkshakes |
| FF6_2 | Fruit juice |
| FF6_3 | Fresh fruit and vegetables |
| FF6_4 | Potato crisps |
| FF6_5 | Red and processed meat |
| FF6_6 | Cakes |
| FF6_7 | Ready meals |
| FF6_8 | Hot takeaways and deliveries |
| FF7 | Some taxes are applied to products to specifically achieve certain goals, either through the revenue raised or by discouraging people to purchase those products and encouraging them to purchase others instead. <br> Thinking about different goals that could |
| FF8 | Would you support or oppose the following… |
| FF8_1 | Making it less expensive for people to choose healthy foods (e.g. fruit and vegetables), and more expensive to choose less healthy foods (e.g. deserts, prepared meals) |
| FF8_2 | Making it less expensive for people to choose healthier options within food groups (e.g. ready meals containing less fat, salt and sugar) and more expensive to choose less healthy options within the same food group |

**A.3. Further descriptive statistics**

**Table A2.** Descriptive statistics for sample political party preferences

|  | | **Weighted Sample** | | **Unweighted**  **Sample** | | **Population**  *%* |
| --- | --- | --- | --- | --- | --- | --- |
|  |  | *N* | *%* | *N* | *%* |  |
| **Vote in 2019 General Elections** | *Con* | 678 | 31.9 | 690 | 32.5 | 43.6 |
|  | *Lab* | 497 | 23.4 | 494 | 23.2 | 32.1 |
|  | *Lib Dem* | 178 | 8.4 | 179 | 8.4 | 11.5 |
|  | *Did not vote* | 480 | 22.59 |  |  |  |
|  | *Do not know* | 45 | 2.12 |  |  |  |
|  | *Skipped/Not asked* | 28 | 1.32 |  |  |  |
| **EU Referendum 2016** | *Remain* | 746 | 35.1 | 861 | 40.5 | 48.11 |
|  | *Leave* | 788 | 37.1 | 775 | 36.5 | 51.89 |
|  | *Did not vote* | 453 | 21.32 |  |  |  |
|  | *Can’t remember* | 36 | 1.69 |  |  |  |

*Notes:* The total sample size is 2,125 adults. Fieldwork was undertaken between 12th - 14th April 2024. The survey was carried out online. The figures have been weighted and are representative of all UK adults (aged 18+). *Source:* YouGov and Office National Statistics (ONS). YouGov Plc did not provide information about the unweighted sample categories for “Did not vote," "Do not know," and "Skipped/Not asked."

**APPENDIX B. Empirical Methodology and Sensitivity Analyses**

**B.1. Empirical Methodology**

To investigate the relationship between individuals’ sociodemographic characteristics and support for unhealthy food taxes, we estimate the following regression:

$y_{ir}=\alpha+\gamma X_{ir}+\emptyset_{r}+\mu_{i} (1)$

Where $y_{i}$ is a set of outcome variables capturing to what extent support a tax on unhealthy foods by person $i.$ These variables are binary regarding the extent of support for taxes on unhealthy food, taking values one if the respondent *"strongly/somewhat supports"* and zero if the respondent *"strongly/somewhat opposes."* Sociodemographic characteristics included in the analysis are age groups, a binary variable capturing high social class (takes one if the respondent’s social class is *ABC1*, and zero if *C2DE.* We also include political options in the last 2019 election and region/country fixed effects. We estimate a logistic fixed-effect mdoel (Logit-FE) as a main empirical methodology, but also a linear probability model (LPM) and an order logistic regression. We provide estimates with robust standard errors, and survey weights are used in all the regressions.

**B.2.** **Sensitivity Analyses**

The following sensitivity analyses have been performed, including (i) a hypothesis test on the statistical differences among the control variables for our two main outcomes, (ii) a multicollinearity test, Variance inflation factor (VIF), and correlation plots, and (iii) linear probability model and ordered logistic regression as a main econometric specification.

1. **Test for supporting tax with subsidy:**

1. **Test for supporting tax on unhealthy foods**

**Table B1.** Multicollinearity test (VIF) among socioeconomic variables

| **Variable** | **VIF** | **1/VIF** |
| --- | --- | --- |
| Age<30 | 1.30 | 0.77 |
| Age 30-44 | 1.50 | 0.66 |
| Age 45-64 | 1.36 | 0.73 |
| Female | 1.02 | 0.98 |
| High Social Class | 1.06 | 0.94 |
| Brexit Party | 2.50 | 0.40 |
| Conservative | 19.01 | 0.05 |
| Green | 3.01 | 0.33 |
| Labour | 16.85 | 0.05 |
| Other | 8.61 | 0.11 |
| Plaid Cymru | 1.38 | 0.72 |
| SNP | 4.31 | 0.23 |
| London | 1.06 | 0.94 |
| Scotland | 1.71 | 0.58 |
| Wales | 1.12 | 0.89 |
| Northern Ireland | 2.88 | 0.34 |

To interpret the results:

- A value of 1 indicates there is no correlation between a given explanatory variable and any other explanatory variables in the model.
- A value between 1 and 5 indicates a moderate correlation between a given explanatory variable and other explanatory variables in the model, but this is often not severe enough to require attention.
- A value greater than 5 indicates a potentially severe correlation between a given explanatory variable and other explanatory variables in the model. In this case, the coefficient estimates and p-values in the regression output are likely unreliable.

**Figure B1.** Linear probability model estimates showing the correlation between support for a tax on unhealthy foods and socioeconomic characteristics

Notes: The number of observations is 1,971 and 1,995, respectively, as those answering ‘do not know’ are not included in both outcome variables. Sample weights are used, and robust standard errors are estimated. Base categories: age between 18-30; male; manual professions (social class grade: C2/D/E); other countries and English regions of the UK.

**Figure B2.** Order logistic model estimates showing the correlation between support for a tax on unhealthy foods and socioeconomic characteristics (outcome variable 1: Tax on unhealthy foods)

Notes: Order logistic regression analysis where outcome 3 means somewhat supports and outcome 5 strongly supports (outcomes 2 and 4 capture somewhat and strongly oppose responses, respectively). Average marginal effects estimates (green/orange dots) are shown. Sample weights are used, and confidence intervals (green/orange lines) are plotted. The number of observations is 1,971, as those answering ‘do not know’ (outcome 1 in the order logit model) are not included in both outcome variables. Sample weights are used, and robust standard errors are estimated. Base categories: age between 18-30; male; manual professions (social class grade: C2/D/E); other countries and English regions of the UK.

**Figure B3.** Order logistic model estimates showing the correlation between support for a tax on unhealthy foods to make healthier food cheaper and socioeconomic characteristics

Notes: Order logistic regression analysis where outcome 3 means somewhat supports and outcome 5 strongly supports (outcomes 2 and 4 capture somewhat and strongly oppose responses, respectively). Average marginal effects estimates (green/orange dots) are shown. Sample weights are used, and confidence intervals (green/orange lines) are plotted. The number of observations is 1,995, as those answering ‘do not know’ (outcome 1 in the order logit model) are not included in both outcome variables. Sample weights are used, and robust standard errors are estimated. Base categories: age between 18-30; male; manual professions (social class grade: C2/D/E); other countries and English regions of the UK.

**Table B2.** Main logistic regression estimates, including controls for self-reported knowledge of food taxes

|  | **Tax on unhealthy food** | | **To make healthier food cheaper** | |
| --- | --- | --- | --- | --- |
| *Average Marginal Effects* | *(1)* | *(2)* | *(3)* | *(4)* |
| Age 30-44 | -0.046  (0.038) | -0.044  (0.038) | -0.080**  (0.035) | -0.082**  (0.035) |
| Age 45-64 | -0.010  (0.039) | -0.001  (0.038) | -0.061*  (0.036) | -0.059  (0.036) |
| Age>64 | 0.009  (0.038) | 0.018  (0.038) | -0.093***  (0.034) | -0.094***  (0.019) |
| Female | 0.022  (0.023) | 0.014  (0.023) | 0.090***  (0.020) | 0.086***  (0.019) |
| High social class | 0.102***  (0.023) | 0.106***  (0.023) | 0.091***  (0.019) | 0.095***  (0.019) |
| London | 0.100**  (0.039) | 0.107***  (0.039) | 0.063**  (0.032) | 0.060*  (0.032) |
| Self-reported knowledge of food taxes, including VAT and SDIL (Based category: poor knowledge) | | | | |
| VAT: Little knowledgeable | -0.112  (0.073) |  | 0.043  (0.068) |  |
| VAT: Knowledgeable | 0.012  (0.068) |  | 0.094  (0.063) |  |
| VAT: Very knowledgeable | -0.033  (0.070) |  | 0.056  (0.056) |  |
| SDIL: Little knowledgeable |  | 0.005  (0.037) |  | 0.039  (0.031) |
| SDIL: Knowledgeable |  | 0.065**  (0.032) |  | 0.015  (0.027) |
| SDIL: Very knowledgeable |  | -0.008  (0.039) |  | -0.024  (0.030) |
| R squared | 0.018 | 0.016 | 0.032 | 0.032 |
| Number of observations | 1,971 | 1,971 | 1,995 | 1,995 |

Notes: ^*^ *p* < 0.1, ^**^ *p* < 0.05, ^***^ *p* < 0.01. Average marginal effects. The number of observations is 1,971 and 1,995, respectively, as those answering ‘do not know’ are not included in both outcome variables. Sample weights are used, and robust standard errors are estimated. Base categories: age between 18-30; male; manual professions (social class grade: C2/D/E); other countries and English regions of the UK. Self-reported knowledge of taxes on food and beverages are defined as follows: poor knowledge: "I have never heard of it and don’t know anything about what foods it is added to"; Little knowledgeable: "I have heard of it, but don't know anything about what foods it is added to"; Knowledgeable: "I know what it is, but know little about what foods it is added to"; and Very knowledgeable: "I know what it is, and the foods it is added to". VAT= Value-added tax; SDIL= Soft Drinks Industry Levy.

**APPENDIX C. Further Tables and Figures**

**Table C1.** ANOVA Post-hoc Bonferroni multiple-comparison test for significance difference in supporting a tax on unhealthy food by professional categories

|  | **Tax on unhealthy food** | | | | |
| --- | --- | --- | --- | --- | --- |
| *Row Mean-*  *Col Mean* | *(1)*  *Professionals* | *(2)*  *Middle -management* | *(3)*  *Skilled manual workers* | *(4)*  *Junior-management* | *(5)*  *Unskilled manual work* |
| *Middle -management* | -2.576 (1.000) |  |  |  |  |
| *Skilled manual workers* | -11.406 (0.028) | -8.830 (0.144) |  |  |  |
| *Junior-management* | -13.295  (0.012) | -10.718 (0.062) | -1.888  (1.000) |  |  |
| *Unskilled manual work* | -18.847 (0.000) | -16.271 (0.001) | -7.440  (0.752) | -5.552  (1.000) |  |
| *Unemployed/State benefits* | -21.682 (0.000) | -19.106 (0.000) | -10.275  (0.206) | -8.387  (0.885) | -2.834  (1.000) |

Notes: Bonferroni-adjusted means and significance (in brackets) of the difference in supporting are reported. The number of observations is 2,125; the base category includes the following responses: Strongly/somewhat opposed/Don't know. The support category includes responses that Strongly/somewhat support. Socioeconomic status groups include the following professions: Professionals (grade A: Professionals; very senior managers in business; top-level civil servants); Middle-management (Grade B: Middle-management executives/Principal officers/Top management or owners of small businesses); Junior-management (Grade C1:Junior management/varied responsibilities and educational requirements); Skilled manual workers (Grade C2:Skilled manual workers/Manual workers with responsibility for other people); Unskilled manual workers (Grade D: Semi-skilled and unskilled manual workers, apprentices and trainees of skilled workers); and Unemployed/State benefits (Grade E: Long-term recipients of state benefits/Unemployed/Off sick/casual workers). Sample weights are used.

**Table C2.** ANOVA Post-hoc Bonferroni multiple-comparison test for significance difference in supporting a tax on unhealthy food to make healthier food cheaper by professional categories

|  | **To make healthier food cheaper** | | | | |
| --- | --- | --- | --- | --- | --- |
| *Row Mean-*  *Col Mean* | *(1)*  *Professionals* | *(2)*  *Middle -management* | *(3)*  *Skilled manual workers* | *(4)*  *Junior-management* | *(5)*  *Unskilled manual work* |
| *Middle -management* | 1.311  (1.000) |  |  |  |  |
| *Skilled manual workers* | -2.541 (1.000) | -3.853 (1.000) |  |  |  |
| *Junior-management* | -9.729  (0.059) | -11.041 (0.007) | -7.187  (0.149) |  |  |
| *Unskilled manual work* | -7.595 (0.617) | -8.907 (0.172) | -5.054  (1.000) | 2.133  (1.000) |  |
| *Unemployed/State benefits* | -18.086 (0.000) | -19.398 (0.000) | -15.545  (0.000) | -8.357  (0.390) | -10.491 (0.149) |

Notes: Bonferroni-adjusted means and significance (in brackets) of the difference in supporting are reported. The number of observations is 2,125; the base category includes the following responses: Strongly/somewhat opposed/Don't know. The support category includes responses that Strongly/somewhat support. Socioeconomic status groups include the following professions: Professionals (grade A: Professionals; very senior managers in business; top-level civil servants); Middle-management (Grade B: Middle-management executives/Principal officers/Top management or owners of small businesses); Junior-management (Grade C1:Junior management/varied responsibilities and educational requirements); Skilled manual workers (Grade C2:Skilled manual workers/Manual workers with responsibility for other people); Unskilled manual workers (Grade D: Semi-skilled and unskilled manual workers, apprentices and trainees of skilled workers); and Unemployed/State benefits (Grade E: Long-term recipients of state benefits/Unemployed/Off sick/casual workers). Sample weights are used.

**Table C3.** Main logistic regression specification estimates, including political preference variables

|  | **Tax on unhealthy food** | | **To make healthier food cheaper** | |
| --- | --- | --- | --- | --- |
| *Average Marginal Effects* | *(1)*  *Logit model* | *(2)*  *Logit model with political preference* | *(3)*  *Logit model* | *(4)*  *Logit model with political preference* |
| Age 30-44 | -0.041  (0.038) | -0.054  (0.039) | -0.080**  (0.035) | -0.085**  (0.036) |
| Age 45-64 | 0.001  (0.039) | -0.005  (0.040) | -0.057  (0.036) | -0.049  (0.037) |
| Age>64 | 0.023  (0.038) | 0.029  (0.041) | -0.088**  (0.034) | -0.064*  (0.037) |
| Female | 0.016  (0.023) | 0.010  (0.023) | 0.088***  (0.020) | 0.081***  (0.020) |
| High social class | 0.107***  (0.023) | 0.093***  (0.023) | 0.094***  (0.019) | 0.084***  (0.020) |
| London | 0.103**  (0.041) | 0.096**  (0.039) | 0.065*  (0.035) | 0.056*  (0.033) |
| Political preferences (Based category: Conservative) | | | | |
| Did not vote |  | -0.002  (0.035) |  | 0.037  (0.030) |
| Don't know |  | 0.130  (0.081) |  | 0.133**  (0.062) |
| Brexit Party |  | -0.169**  (0.084) |  | -0.031  (0.087) |
| Green |  | 0.280***  (0.070) |  | 0.175***  (0.051) |
| Labour |  | 0.094***  (0.034) |  | 0.123***  (0.026) |
| Liberal Democratic |  | 0.043  (0.044) |  | 0.030  (0.040) |
| Other |  | 0.053  (0.070) |  | 0.037  (0.063) |
| Plaid Cymru |  | -0.096  (0.180) |  | 0.136  (0.056) |
| Scottish National Party |  | 0.083  (0.074) |  | 0.112**  (0.056) |
| R squared | 0.013 | 0.023 | 0.030 | 0.043 |
| Number of observations | 1,971 | 1,971 | 1,995 | 1,995 |

Notes: ^*^ *p* < 0.1, ^**^ *p* < 0.05, ^***^ *p* < 0.01. Average marginal effects. The number of observations is 1,971 and 1,995, respectively, as those answering ‘do not know’ are not included in both outcome variables. Sample weights are used, and robust standard errors are estimated. Base categories: age between 18-30; male; manual professions (social class grade: C2/D/E); other countries and English regions of the UK. R squared is McFadden’s Pseudo R squared.

**Figure C1**. Self-reported knowledge of taxes on food and beverages in the United Kingdom: share of respondents


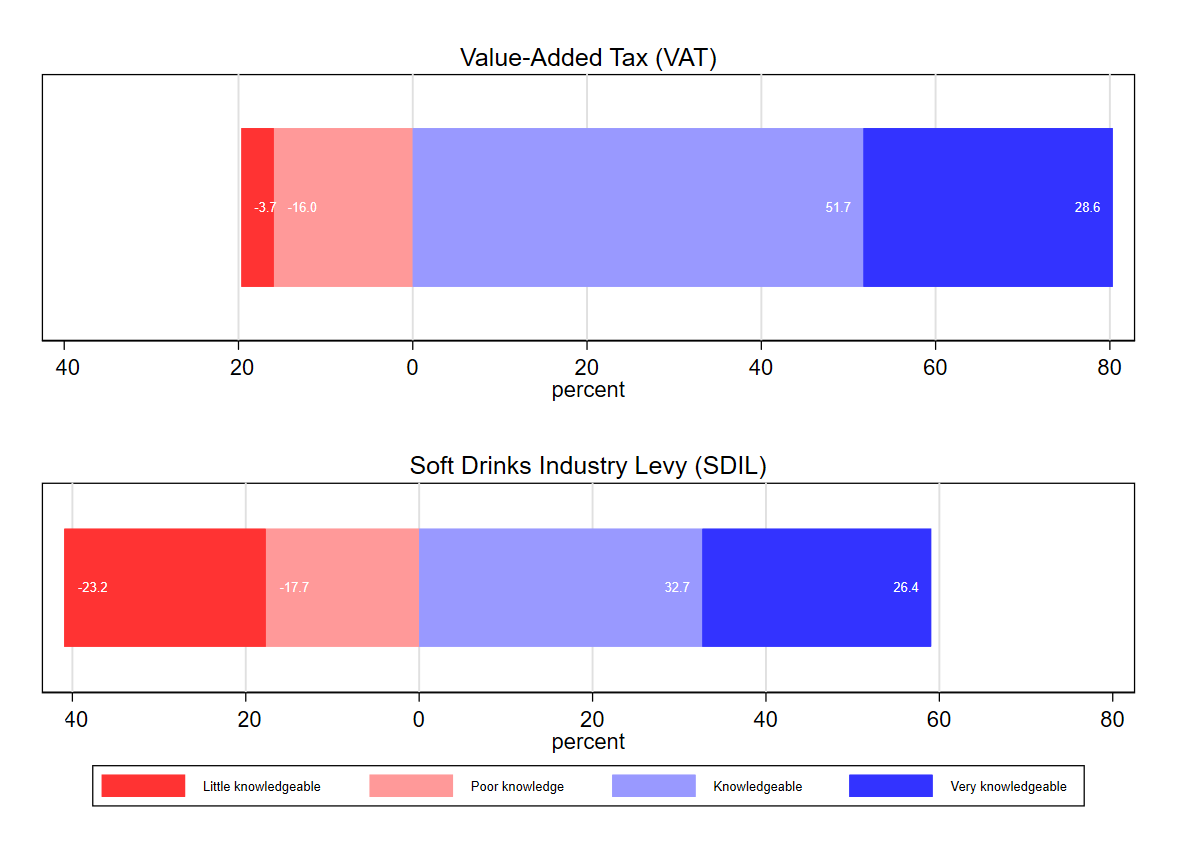


*Notes*: Total sample size are 2,125 adults. Fieldwork was undertaken between 12th - 14th April 2024. Self-reported knowledge of taxes on food and beverages are defined as follows: poor knowledge: "I have never heard of it and don’t know anything about what foods it is added to"; Little knowledgeable: "I have heard of it, but don't know anything about what foods it is added to"; Knowledgeable: "I know what it is, but know little about what foods it is added to"; and Very knowledgeable: "I know what it is, and the foods it is added to". We are using average weights. Sample weights are used.

**Figure C2.** Knowledge about food groups which have VAT applied: Share of correct answers in dark blue


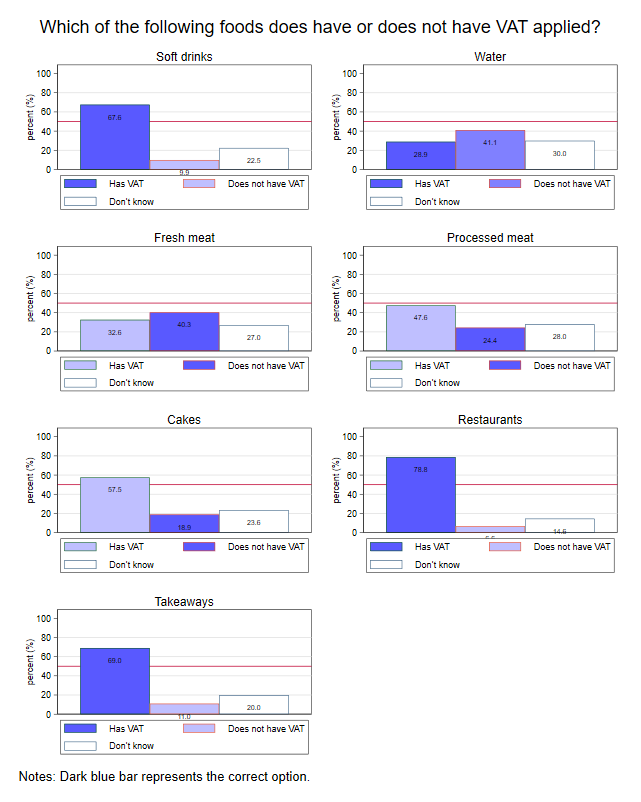


*Notes:* The total sample size is 2,125 adults. Fieldwork was undertaken between 12th - 14th April 2024. Sample weights are used.

**Figure C3.** Share of respondents support a higher tax on unhealthy foods by age groups

*Notes:* The total sample size is 2,125 adults. Fieldwork was undertaken between 12th - 14th April 2024. The base category includes the following responses: Strongly/somewhat opposed/Don't know. The support category includes responses that Strongly/somewhat support. Sample weights are used.

**Figure C4.** Share of respondents who support a higher tax on unhealthy foods by political party


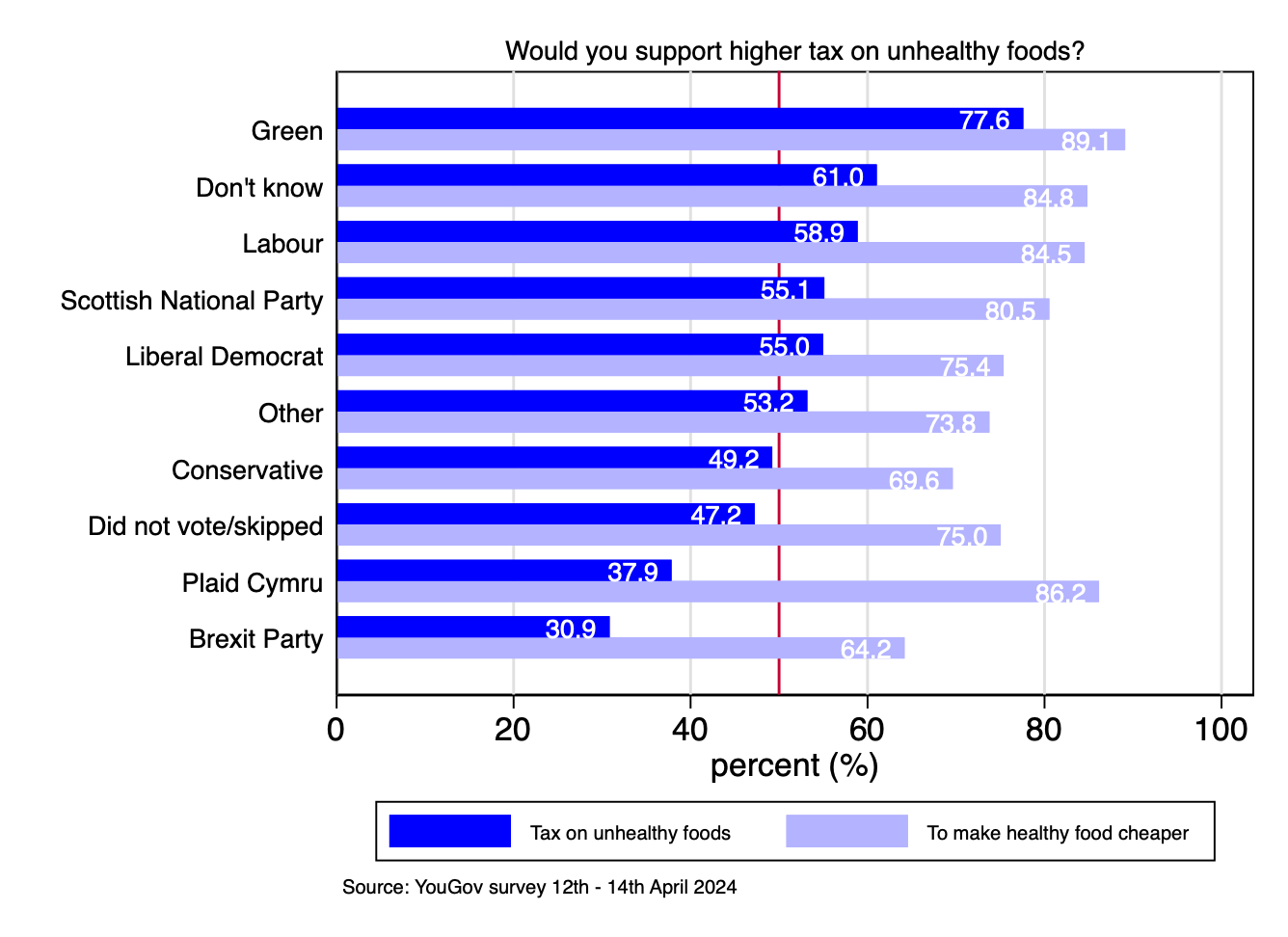


*Notes*: Total sample size are 2,125 adults. Fieldwork was undertaken between 12th - 14th April 2024. The sample size by “Do not know” contains only 45 observations, and “did not vote or skipped” contains 508 observations. Sample weights are used.

**Figure C5.** Share of respondents who agree (somewhat to strongly) that “would you support a higher tax on unhealthy foods?” or “Would you support a higher tax on unhealthy foods to make healthy food cheaper” by regions


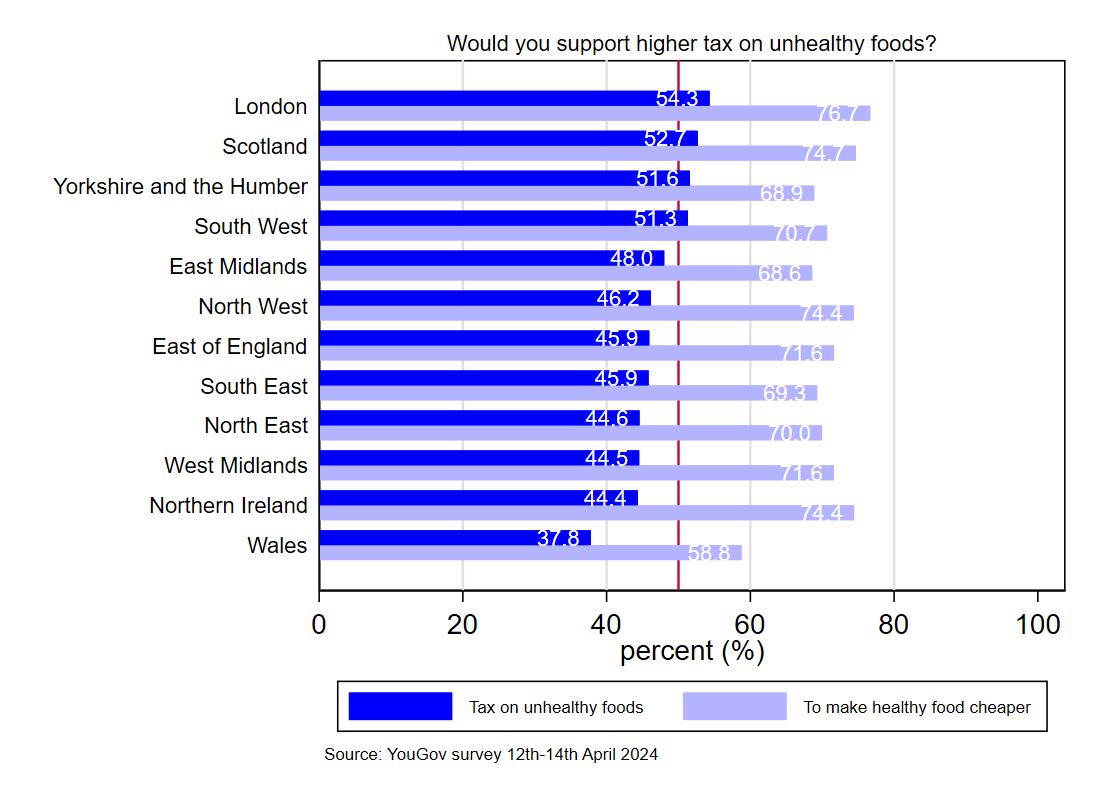


Notes: The total sample size is 2,125 adults; the base category includes the following responses: Strongly/somewhat opposed/Don't know. The support category includes responses that Strongly/somewhat support. Sample weights are used.

**Figure C6.** Support for tax on unhealthy foods between different food categories

Notes: The total sample size is 2,125 adults. Sample weights are used.

**Figure C7.** Support for tax on unhealthy foods within foods in the same category

Notes: The total sample size is 2,125 adults. Sample weights are used.

1. The terms and conditions that respondents are required to agree to when becoming a panellist can be found on the website (<https://account.yougov.com/gb-en/account/terms-and-conditions#terms>). The two policies we ask people to consent to are below, and the form is in the following link: <https://account.yougov.com/gb-en/account/privacy-policy>. [↑](#footnote-ref-2)
